# Emergence of plasmid-borne *tet*(X4) resistance gene in clinical isolate of eravacycline- and omadacycline-resistant *Klebsiella pneumoniae* ST485

**DOI:** 10.1128/spectrum.00496-24

**Published:** 2024-07-23

**Authors:** Xiaojing Liu, Yi Liu, Xiaohan Ma, Ruyan Chen, Chenyu Li, Hongxin Fu, Yu Yang, Kexin Guo, Xiaoping Zhang, Ruishan Liu, Hao Xu, Junfei Zhu, Beiwen Zheng

**Affiliations:** 1 Shandong First Medical University and Shandong Academy of Medical Sciences, Jinan, China; 2 Jinan Microecological Biomedicine Shandong Laboratory, Jinan, China; 3 Collaborative Innovation Center for Diagnosis and Treatment of Infectious Diseases, State Key Laboratory for Diagnosis and Treatment of Infectious Diseases, the First Affiliated Hospital, College of Medicine, Zhejiang University, Hangzhou, China; 4 The First Affiliated Hospital of Beihua University, Jilin, China; 5 Department of Laboratory Medicine, The First Affiliated Hospital of Zhengzhou University, Zhengzhou, China; 6 School of Basic Medical Sciences, Zhejiang Chinese Medical University, Hangzhou, China; 7 Department of Respiratory Medicine, Taizhou Central Hospital, Taizhou, China; 8 Research Units of Infectious Diseases and Microecology, Chinese Academy of Medical Sciences, Beijing, China; Ross University School of Veterinary Medicine, Basseterre, Saint Kitts and Nevis

**Keywords:** *Klebsiella pneumoniae*, *tet*(X4), omadacycline, eravacycline, ST485, IS*Vsa3*

## Abstract

Omadacycline and eravacycline are gradually being used as new tetracycline antibiotics for the clinical treatment of Gram-negative pathogens. Affected by various tetracycline-inactivating enzymes, there have been reports of resistance to eravacycline and omadacycline in recent years. We isolated a strain carrying the mobile tigecycline resistance gene *tet*(X4) from the feces of a patient in Zhejiang Province, China. The strain belongs to the rare ST485 sequence type. The isolate was identified as *Klebsiella pneumoniae* by matrix-assisted laser desorption/ionization time-of-flight mass spectrometry (MALDI-TOF MS). The MICs of antimicrobial agents were determined using either the agar dilution method or the micro broth dilution method. The result showed that the isolate was resistant to eravacycline (MIC = 32 mg/L), omadacycline (MIC > 64 mg/L), and tigecycline (MIC > 32 mg/L). Whole-genome sequencing revealed that the *tet*(X4) resistance gene is located on the IncFII(pCRY) conjugative plasmid. *tet*(X4) is flanked by IS*Vsa3*, and we hypothesize that this association contributes to the spread of the resistance gene. Plasmids were analyzed by S1-nuclease pulsed-field gel electrophoresis (S1-PFGE), Southern blotting, and electrotransformation experiment. We successfully transferred the plasmid carrying *tet*(X4) to the recipient bacteria by electrotransformation experiment. Compared with the DH-5α, the MICs of the transformant L3995-DH5α were increased by eight-fold for eravacycline and two-fold higher for omadacycline. Overall, the emergence of plasmid-borne *tet*(X4) resistance gene in a clinical isolate of *K. pneumoniae* ST485 underscores the essential requirement for the ongoing monitoring of *tet*(X4) to prevent and control its further dissemination in China.

**IMPORTANCE:**

There are still limited reports on *Klebsiella pneumoniae* strains harboring tetracycline-resistant genes in China, and *K. pneumoniae* L3995hy adds a new example to those positive for the *tet*(X4) gene. Importantly, our study raises concerns that plasmid-mediated resistance to omadacycline and eravacycline may spread further to a variety of ecological and clinical pathogens, limiting the choice of medication for extensively drug-resistant bacterial infections. Therefore, it is important to continue to monitor the prevalence and spread of *tet*(X4) and other tetracyclines resistance genes in *K. pneumoniae* and diverse bacterial populations.

## INTRODUCTION

*Klebsiella pneumoniae*, an opportunistic pathogen, ranks among the most common causes of both hospital- and community-acquired infections. It often manifests as respiratory, urinary tract infections, neurologic, intra-abdominal, and bloodstream infections. The escalation of antibiotic resistance stands as one of the paramount threats to global health (1). The prevalence of carbapenem-resistant Enterobacteriaceae (CRE) is rapidly increasing and, with the global spread of plasmid-mediated mobile colistin resistance (MCR), tigecycline is now one of the last options for the treatment of severe infections caused by *K. pneumoniae* (2, 3). With the inevitable emergence of tigecycline resistance since its introduction to clinical treatment in 2005, this undoubtedly poses a significant challenge for the treatment of CRKP (4). In 2018, two novel tetracyclines were approved by the US Food and Drug Administration (FDA): eravacycline and omadacycline. These two third-generation tetracyclines showed lower adverse effects and better antibacterial activity compared with tigecycline, respectively, and are therefore considered to be the most appropriate treatment for patients with severe community-sourced XDR bacterial infections in patients with severe community origin (5, 6).

Chromosomal mutations, overexpression of the efflux pump, or mutations in the ribosome, have long been recognized as the primary mechanisms leading to resistance to tigecycline in *K. pneumoniae* (7, 8). Mutations in *acrR*, *ramR*, *plsC*, *rpsJ*, *trm*, *tet*(A), and *tet*(M) were found to decrease tigecycline sensitivity (9, 10). The plasmid-borne *tet*(X) resistance gene, encoding a flavin monooxygenase, represents a novel mechanism of resistance to a class of tigecycline (11). Indeed, *tet*(X) effectively degrades almost all tetracycline antibiotics *in vitro*, mediating high levels of resistance to tigecycline antibiotics (12, 13). Various *tet*(X) gene variants mediate different levels of tigecycline resistance, with the plasmid-mediated expression product of the *tet*(X4) gene belonging to the core members of the degradative enzyme machinery (5, 9, 14). Except for the discovery of a strain of *K. pneumoniae* carrying the *tet*(X4) resistance gene by Zhai et al. in Beijing in 2019 (15), clinical carriage of the *tet*(X4) resistance gene is currently seen mainly in bacteria such as *Escherichia coli. K. pneumoniae* ST485 is a rare isolate. Previously, Kang screened 24/27 strains of ST-type *K. pneumoniae* in the ICU environment, suggesting that no major outbreak of ST485 transmission has developed in the country (16).

Given the emergence of clinical *tet*(X4)-carrying *K. pneumoniae*, it is urgent to investigate the genetic environment and transmission mechanisms of the *tet*(X4) resistance gene. This study reports the identification and comprehensive characterization of multidrug-resistant *K. pneumoniae* isolated from patient feces. This strain carries the *tet*(X4) resistance gene on a transferable plasmid that confers resistance to omadacycline and eravacycline.

## RESULTS

### Isolation and characterization of *tet*(X4)-carrying *K. pneumoniae* strain L3995hy

Following admission to the hospital in 2021 with a headache and fever, the male patient received treatment with ceftriaxone and meropenem. After being diagnosed with tuberculous meningitis (TBM) and receiving anti-tuberculosis treatment, he was later discharged from a Grade-A tertiary hospital in Zhejiang Province following hospitalization for an intracranial infection. Strain L3995hy was isolated from the feces of this male patient and identified as *K. pneumoniae*. Clinical reports showed it was resistant to tigecycline, eravacycline, and omadacycline. PCR revealed the presence of a *tet*(X4) gene.

### Bacterial electrotransformation

The transformant L3995-DH5α was selected and identified as *E. coli* by MALDI-TOF MS. They were confirmed to be *tet*(X4)-positive by PCR. Consequently, it was demonstrated that the plasmid carrying *tet*(X4) from the donor L3995hy could transfer into *E. coli* DH5α.

### AST of *K. pneumoniae* L3995hy

Antimicrobial susceptibility analysis (Table 1) showed that strain L3995hy is highly resistant to tetracycline antibiotics (tigecycline, tetracycline, doxycycline, and minocycline), including resistance to the newly FDA-approved tetracycline antibiotics eravacycline and omadacycline. In addition, the strain exhibited resistance to aztreonam, ceftriaxone, cefotaxime, ceftazidime, and piperacillin-tazobactam. It showed sensitivity to fosfomycin, imipenem, ceftazidime-avibactam, and colistin. Moreover, transformant L3995-DH5α demonstrated identical antibiotic resistance to omadacycline, minocycline, doxycycline, and tetracycline as L3995hy but showed an intermediate response to tigecycline.

**TABLE 1:** Susceptibility of *K. pneumoniae* L3995hy and its transformant to commonly used antibiotics^*a*^

Antibiotic	MIC value (mg/L)		
	L3995hy	DH-5α	L3995-DH5α
Tigecycline	>32 (R)	1 (S)	4 (I)
Eravacycline	32	0.25 (S)	2
Omadacycline	>64 (R)	8 (I)	16 (R)
Minocycline	128 (R)	2 (S)	16 (R)
Doxycycline	64 (R)	2 (S)	16 (R)
Tetracycline	128 (R)	2 (S)	128 (R)
Aztreonam	>128 (R)	0.125	0.25 (S)
Ceftriaxone	>128 (R)	≤0.03 (S)	0.125 (S)
Cefotaxime	>128 (R)	≤0.03 (S)	0.5 (S)
Ceftazidime	>128 (R)	0.03 (S)	1 (S)
Piperacillin-tazobactam	64/4 (R)	8 (S)	8 (S)
Amoxicillin-clavulanate	32/16 (R)	4 (S)	16 (I)
Cefepime	>128 (R)	0.015 (S)	0.03 (S)
Levofloxacin	64 (R)	0.03 (S)	0.25 (S)
Ciprofloxacin	64 (R)	0.015 (S)	0.125 (S)
Chloramphenicol	>128 (R)	4 (S)	16 (I)
Fosfomycin	64 (S)	≤0.25 (S)	0.25 (S)
Imipenem	1 (S)	0.125 (S)	0.125 (S)
Meropenem	2 (I)	0.03 (S)	0.03 (S)
Ceftazidime-avibactam	1/4 (S)	≤0.03 (S)	≤0.03 (S)
Colistin	1 (I)	0.25 (S)	0.25 (S)

^*a*^ R, resistant; S, susceptible; I, intermediate.

### Genomic characteristics of L3995hy

Table S1 summarizes the genomic characteristics of *K. pneumoniae* L3995hy. WGS identified *K. pneumoniae* L3995hy as belonging to ST485. The genome of *K. pneumoniae* L3995hy contains a 5,260,925 bp circular chromosome with an average GC content of 57.4%. It also comprises six plasmid sequences with various sizes ranging from 23,166 bp to 78,154 bp (Table S1). Acquired resistance genes were identified through the analysis of antibiotic resistance genes (ARGs) using ResFinder. Both the chromosome and plasmid of *K. pneumoniae* strain L3995hy harbor ARGs conferring resistance to β-lactams (*bla*_CTX-M-55_, *bla*_TEM-1B_, *bla*_SHV-27_, *bla*_SHV-110_, *bla*_SHV-191_), Fosfomycin (*fosA*), aminoglycosides (*aadA2*, *aadA1*, *aph*[*3'*]*-IIa*, *aph*[*3'*]*-IIa*, *aadA1*), chloramphenicol (*oqxA*, *oqxB*), the quinolones (*qnrS1*, *oqxB*, *oqxA*), and the tetracyclines (*tet*[A], *tet*[X4]). In addition, the chromosome carries virulence genes associated with biofilm formation (*ompA*, *mrkC*, *mrkD*, and *mrkH*), adhesion (*fimH*, *fimF*, and *fimC*), *etc*. (Table S2).

### Characterization of plasmid harboring *tet*(X4)

**Fig 1:**
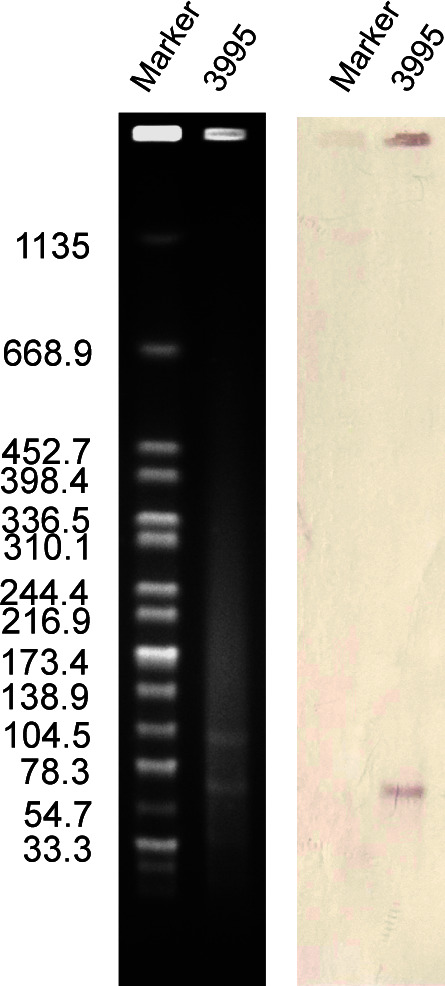
The plasmid size of *K. pneumoniae* L3995hy was determined by S1-PFGE, with *Salmonella enterica* serotype Braenderup H9812 as the size marker. Southern blotting hybridization with a *tet*(X4)-specific probe.

**Fig 2:**
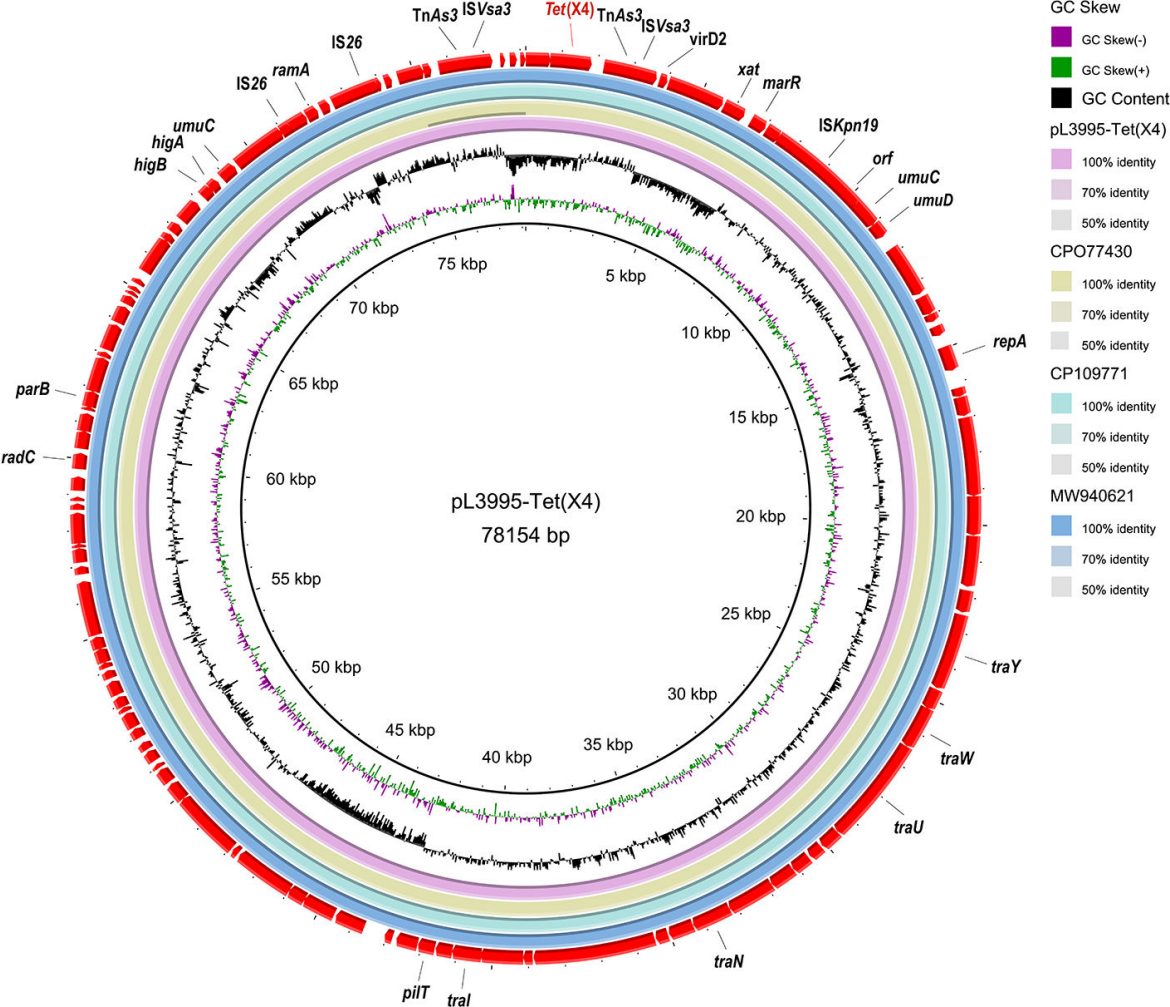
Comparative analysis of plasmids pL3995-*tet*(X4) with pNTT31XS-*tet*(X4) (no.CP077430), pYZ-58-*tet(*X4) (no.CP109771), and pSDP9R-*tet*(X4) (no.MW940621).

**Fig 3:**
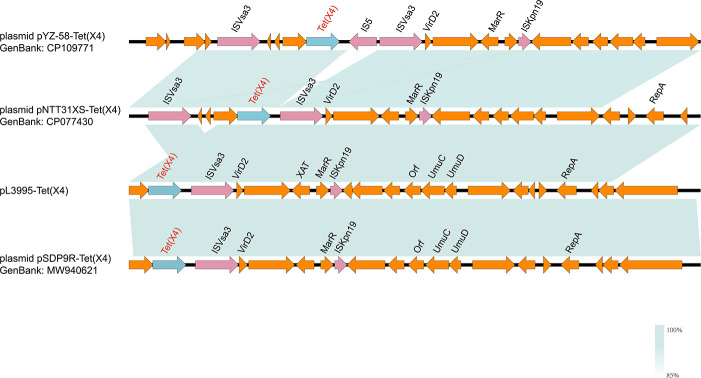
Genomic analyses of plasmid pL3995-*tet*(X4). Open reading frames (ORFs) are indicated by arrows and are denoted according to their presumed function. Blue indicates resistance genes, pink indicates removable element-related genes, and orange indicates other functional genes. Regions with a high degree of homology are shaded in blue.

Based on ResFinder and S1-PFGE results, the *tet*(X4) resistance gene was identified on a 78,154 bp long plasmid (Fig. 1), with its replicon belonging to the IncFII(pCRY) incompatibility group. Subsequently, a BLASTN search was performed using plasmid pL3995-Tet(X4) containing *tet*(X4) as a reference sequence in the NCBI database. By searching the core plasmid region against those in GenBank, the pL3995-Tet(X4) backbone showed 100% query coverage and 99.9% nucleotide identity to plasmid pNTT31XS-tetX4 from porcine intestinal contents *Klebsiella aerogenes* strain (no. CP077430), plasmid pYZ-58-tetX from pork *K. pneumoniae* strain (no. CP109771), plasmid pSDP9R-tetX4 from pork *Klebsiella* sp. strain (no. MW940621). Analysis of the genetic environment (Fig. 2 and 3) showed that IS*Vsa3* is located upstream and downstream of *tet*(X4).

## DISCUSSION

*K. pneumoniae* is one of the prevalent bacterial pathogens responsible for nosocomial and serious community-acquired infections. The escalating antimicrobial drug resistance it exhibits presents a formidable therapeutic challenge (17, 18). Eravacycline has higher clinical efficacy and better tolerability than tigecycline for abdominal infections caused by common pathogens such as *K. pneumoniae*. Previous studies have found that the minimum inhibitory concentration (mg/L) inhibiting 90% of isolates (MIC_90_) of eravacycline is generally lower than that of tigecycline and omadacycline (19). The gene *tet*(X4), which is one of the most prevalent tigecycline resistance genes, has been detected in *K. pneumoniae* from the environment, animals, and edible meats (20–22). However, clinical isolates of *K. pneumoniae* carrying *tet*(X4) have rarely been reported. In this article, we isolated a strain of *K. pneumoniae* carrying *tet*(X4) from a clinical fecal sample, which is highly resistant to all tetracycline antibiotics. We successfully transferred the plasmid carrying *tet*(X4) to the recipient bacteria by electrotransformation experiment. Whole-genome sequencing (WGS) analysis revealed that insertion sequences IS*Vsa3* both upstream and downstream of *tet*(X4) contributed to the transmission of drug-resistant genes. This differs from the common IS*CR2-tet*(X4)-IS*CR2* sequence associated with *tet*(X4).

Previously, the *tet*(X4) gene has been identified on various plasmid types, such as ColE2-like, IncQ, IncX1, IncA/C2, IncFII, IncFIB, among others. Notably, the IncX1-type plasmid emerges as the predominant vector for the *tet*(X4) gene. The type of replicon carrying *tet*(X4) in isolate L3995hy is identified as IncFII(pCRY). The STs of *K. pneumoniae* carrying *tet*(X4)-resistant genes are diverse, with the dominant clone type isolated from a Chinese pig, ST414-1LV (23). It is worth noting that up to now, we have not detected *tet*(X4) resistance genes in the more popular *K. pneumoniae* STs, such as ST11, ST15, and ST258. In contrast to the more prevalent *K. pneumoniae* STs, L3995hy represents a rare ST485 isolate. Importantly, this marks the initial identification of the *tet*(X4) resistance gene in *K. pneumoniae* ST485.

pL3995-Tet(X4) carries both *tet*(X4) and *tet*(A) genes. In contrast to the majority of plasmids carrying the *tet*(X4) resistance gene, there is an absence of IS*CR2* both upstream and downstream of *tet*(X4). Instead, there are mobile genetic elements (MGEs) (Tn*As3* and IS*Vsa3*) (24). IS*CR2* is commonly located both upstream and downstream of *tet*(X4) and mediates the horizontal transfer of *tet*(X4) resistance gene through roll-over replication. Moreover, it often forms a complex genetic structure with Tn*3* that facilitates the transmission of *tet*(X4) resistance gene. Both IS*Vsa3* and IS*CR2* are IS*91*-like transposases capable of mobilizing resistance genes through rolling circle replication. Our observation implies that the involvement of an expanding array of IS elements is involved in the mobilization of the *tet*(X4) gene. Likewise, we believe that the *tet*(X4) gene can be incorporated into a new plasmid with the assistance of Tn*As3* and IS*Vsa3* insertion sequences. In addition to the downstream of *tet*(X4), the insertion of the IS*26* element is observed in the plasmid housing *tet*(X4). This presence of IS*26* is significant, as IS*26*-mediated translocation has been documented to play a pivotal role in mobilizing antimicrobial resistance genes (15, 24).

Historically, tigecycline resistance in *K. pneumoniae* was attributed mainly to the overexpression of genes encoding the AcrAB-TolC efflux pump, which is controlled by the local repressor *acrR* and global transcriptional activators (25). Various efflux mechanisms are associated with low levels of tigecycline resistance (26), and in the case of L3995hy, it carries the *acrR* and *tet*(A) efflux pump gene on chromosome and plasmid, respectively, contributing to low-level resistance to tigecycline. Antimicrobial resistance sensitivity demonstrated that L3995hy exhibited high resistance to all tetracyclines, including the recently FDA-approved eravacycline and omadacycline. Compared with the DH-5α, MICs of the transformant L3995-DH5α were increased by eight-fold for eravacycline, two-fold higher for omadacycline, and ranged from four- to 64-fold higher for tetracyclines such as tigecycline. This suggests that a combination of efflux mechanisms and drug-resistance genes may exert a synergistic effect on resistance (27).

**Fig 4:**
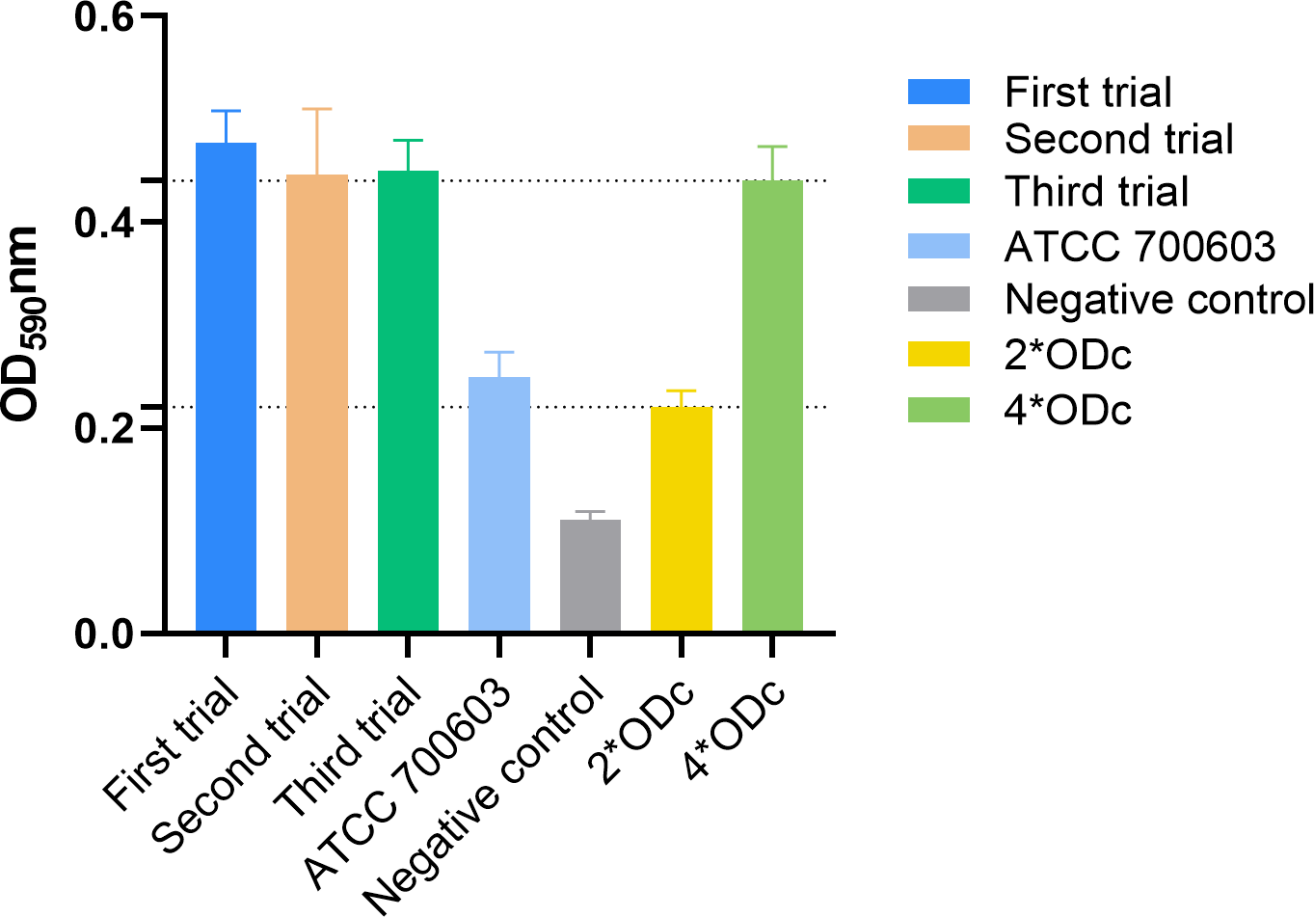
The biofilm biomass of *K. pneumoniae* L3995hy was compared with that of the control strains. ATCC 700603 served as a reference control, while LB broth was employed as the negative control. The critical value, ODc, represents the mean optical density (OD) of the negative control.

Biofilms play a key role in expressing resistance and virulence phenotypes. Biofilm formation assays showed that ATCC 700603 had moderate biofilm formation ability (2*ODc <ODs ≤ 4*ODc) (28), and *K. pneumoniae* L3995hy had strong biofilm formation ability (ODs > 4*ODc) (Fig. 4), suggesting that it has some ability to adhere and colonize. This may mean that this *K. pneumoniae* ST485 strain has the ability to cause clinical challenges. OriTfnder results indicated the absence of the *oriT*, *Relaxase*, T4CP, and T4SS in Plasmid3, Plasmid4, Plasmid5, and Plasmid6. The lack of conjugated transfer gene regions in these plasmid backbones is consistent with the fact that in *vitro* conjugation experiments were not successful (29). In conclusion, given the previous experience of rapid dissemination of carbapenem-resistant plasmids (*bla*_KPC-2_ or *bla*_NDM-1_ plasmids) and colistin-resistant plasmids (*mcr* plasmids), there is a strong suspicion that the emergence of a transmissible tigecycline-resistant plasmid (*tet*[X4]) will significantly contribute to the development of global pan-drug resistance.

## MATERIALS AND METHODS

### Sample collection and bacterial culture

In 2021, a tigecycline-resistant strain of *K. pneumoniae* (strain L3995hy) was isolated from a fecal sample of a male inpatient at a tertiary care hospital in Zhejiang Province, China. The sample was incubated on MacConkey agar plates at 37°C for 18–24 hours. Subsequently, the strain and resistance genes in the isolate were identified by matrix-assisted laser desorption ionization time-of-flight mass spectrometry and PCR amplification.

### Location of *tet*(X4) gene and transferability of plasmids carrying *tet*(X4)

The number and size of the plasmid of *K. pneumoniae* L3995hy were determined with the S1 nuclease pulsed-field gel cataphoresis (S1-PFGE) method, as described previously (30). In addition, the location of the *tet*(X4) gene was determined according to Southern blotting and hybridization with a digoxigenin-labeled *tet*(X4) specific probe. *Salmonella* strain H9812 was used as a control strain and size marker (31). The recombinant vector was transferred into *E. coli* DH5α by the electrotransformation method as described previously (5). Plasmid extraction was performed using the QIAGEN Large-Contruct Kit, and the extracted plasmid was electrotransformed into receptive *E. coli* DH5α by voltage shock, after incubation in SOC medium, it was uniformly applied to a drug-sensitive plate containing tigecycline. Finally, *tet*(X4) was verified by PCR and MALDI-TOF/MS performed strain identification.

### Antimicrobial susceptibility testing

Antimicrobial susceptibility testing (AST) was performed for tetracycline, β-lactam, aminoglycoside, and quinolone antibiotics using either the agar dilution method or the micro-broth dilution method. Polymyxins were interpreted according to the European Committee on Antimicrobial Susceptibility Testing (EUCAST) (https://www.eucast.org/), and tigecycline, omadacycline, and eravacycline were interpreted according to FDA definitions (Tigecycline—Injection products | FDA; Omadacycline Injection and Oral Products | FDA; Eravacycline - Injection Products | FDA). The remaining antibiotics were interpreted according to Clinical and Laboratory Standards Institute (CLSI) standards, and *E. coli* ATCC 25922 was used as a quality control.

### Biofilm formation experiment

According to the tests described in earlier studies, the formation of biofilm was assessed (32). First, the bacteria were inoculated in LB broth for overnight incubation, 200 μL of 0.5 McFarland standard turbidity suspension was added to a 96-well plate, and three well replicates were done for each sample. After standing overnight incubation at 37°C, the plate was washed three times with PBS to eliminate all non-adherent bacteria, then fixed with methanol and stained by adding 150 μL of 0.1% crystal violet solution to each well. After washing three times with PBS and discarding the washing solution, 100 µL of DMSO was added to dissolve the crystal violet attached to the biofilm, followed by incubation for 5 minutes. The OD (optical density) was measured at 590 nm. Three replicate experiments were performed. The standard strain ATCC 700603 was used as a control. LB broth was used as a negative control. ODs ≤ ODc, 2*ODc < ODs ≤ 4*ODc, and ODs > 4*ODc, indicating no biofilm formation ability, moderate biofilm formation ability, and strong biofilm formation ability of bacterium, respectively.

### Whole-genome sequencing and bioinformatics analysis

Genomic DNA was extracted by using a Bacterial DNA Kit (QIAGEN, Hilden, Germany). Following that, the DNA was sequenced to acquire data using both the Illumina NovaSeq 6000 (Illumina, San Diego, CA, USA) and Oxford Nanopore platforms (Oxford Nanopore Technologies, Oxford, United Kingdom) to obtain the strain’s final, whole genome sequencing, sequenced segments were assembled using Unicycler v0.4.7. RAST 2.0 (http://rast.nmpdr.org) was used to annotate it after that. By using the ISfinder database, insertion elements (ISs) were found. Acquired antibiotic resistance genes (ARGs) and plasmid incompatibility types were identified using the ResFinder (https://cge.cbs.dtu.dk/services/ResFinder/) and Plasmid Finder (https://cge.cbs.dtu.dk/services/PlasmidFinder/) databases. Multilocus sequence typing (MLST) was performed on the tigecycline-resistant *K. pneumoniae* isolates by amplifying and sequencing seven housekeeping genes (*gapA*, *infB*, *mdh*, *pgi*, *phoE*, *rpoB*, and *tonB*) according to a previously described protocol. Sequence types (STs) were assigned using the online database (http://pubmlst.org/ecloacae). Using oriTfinder, the source of transfers in the DNA sequences of bacterial mobile genetic elements was found (https://tool-mml.sjtu.edu.cn/oriTfinder/oriTfinder.html). VFDB.1 was used to find the virulence factors. BLASTN was used to compare plasmid sequences with the GenBank database (https://blast.ncbi.nlm.nih.gov/blast.cgi). Finally, the circular image of plasmid comparison and the comparative map of the genetic environment surrounding the *tet*(X4) gene were plotted by BLAST Ring Image Generator (BRIG) and Easyfig, respectively.

## Data Availability

The genome sequencing data are publicly available at NCBI GenBank under the BioProject accession numbers CP135165-CP135171.

## SUPPLEMENTAL MATERIAL

Supplemental material spectrum.00496-24-s0001.docx Tables S1 and S2.

ASM does not own the copyrights to Supplemental Material that may be linked to, or accessed through, an article. The authors have granted ASM a non-exclusive, world-wide license to publish the Supplemental Material files. Please contact the corresponding author directly for reuse.
