# Supplementary material for: Emergence of plasmid-borne tet(X4) resistance gene in clinical isolate of eravacycline- and omadacycline-resistant Klebsiella pneumoniae ST485
Source: Microbiol Spectr. 2024 Jul 23;12(9):e00496-24. doi: 10.1128/spectrum.00496-24 (PMC11370244; doi:10.1128/spectrum.00496-24)
Supplement: Supplemental material — Tables S1 and S2. [file spectrum.00496-24-s0001.docx]

**TABLE S1** Features of chromosome and plasmids harbored by *K. pneumoniae* L3995hy

| Plasmid | Total number of bases (bp) | G+C content (%) | plasmid replicon type | Resistance genes | Accession numbers |
| --- | --- | --- | --- | --- | --- |
| Chromosomes | 5,260,925 | 57.4 | NA | fosA, acrR, cmlA1, OqxA, QqxB, blaSHV-27,  blaSHV-110, blaSHV-191,qacL | CP135165 |
| Contig 3 | 78,154 | 52.9 | IncFII(pCRY) | tet(X4), tet(A) | CP135167 |
| Contig 4 | 45,080 | 52.8 | IncR/IncN | aadA2, qnrS1, sul3, dfrA12, aadA1, mef(B) | CP135168 |
| Contig 5 | 24,680 | 50.7 | NA | aph(3')-Iia, TEM-1B, blaCTX-M-55,  dfrA14, dfrA12 | CP135169 |
| Contig 6 | 23,166 | 48.1 | IncX1 | TEM-1B, blaCTX-M-55 | CP135170 |
| Contig 7 | 2,987 | / | Col440I | / | CP135171 |

Note: NA, is not applicable.

**TABLE S2** Virulence factors harbored by *K. pneumoniae* L3995hy.

| VFclass | Virulence factors | Related genes |
| --- | --- | --- |
| Adherence | Type 3 fimbriae | *mrkA* |
|  |  | *mrkB* |
|  |  | *mrkC* |
|  |  | *mrkD* |
|  |  | *mrkF* |
|  |  | *mrkH* |
|  |  | *mrkI* |
|  |  | *mrkJ* |
|  | Type I fimbriae | *fimA* |
|  |  | *fimB* |
|  |  | *fimC* |
|  |  | *fimD* |
|  |  | *fimE* |
|  |  | *fimF* |
|  |  | *fimG* |
|  |  | *fimH* |
|  |  | *fimI* |
|  |  | *fimK* |
| Efflux pump | AcrAB | *acrA* |
|  |  | *acrB* |
| Iron uptake | Aerobactin | *iutA* |
|  | Ent siderophore | *entA* |
|  |  | *entB* |
|  |  | *entC* |
|  |  | *entD* |
|  |  | *entE* |
|  |  | *entF* |
|  |  | *entS* |
|  |  | *fepA* |
|  |  | *fepB* |
|  |  | *fepC* |
|  |  | *fepD* |
|  |  | *fepG* |
|  |  | *fes* |
|  | Salmochelin | *iroE* |
|  |  | *IroN* |
| Regulation | RcsAB | *rcsA* |
|  |  | *rcsB* |
| Secretion system | T6SS-I | *dotU/tssL* |
|  |  | *hcp/tssD* |
|  |  | *icmF/tssM* |
|  |  | *clpV/tssH* |
|  |  | *ompA* |
|  |  | *sciN/tssJ* |
|  |  | *tssF* |
|  |  | *tssG* |
|  |  | *vasE/tssK* |
|  |  | *vgrG/tssI* |
|  |  | *vipA/tssB* |
|  |  | *vipB/tssC* |
|  | T6SS-II | *clpV* |
|  |  | *dotU* |
|  |  | *icmF* |
|  |  | *impF* |
|  |  | *impH* |
|  |  | *impJ* |
|  |  | *ompA* |
|  |  | *sciN* |
|  |  | *vgrG* |
